# Progress in Confocal Laser Endomicroscopy for Neurosurgery and Technical Nuances for Brain Tumor Imaging With Fluorescein

**DOI:** 10.3389/fonc.2019.00554

**Published:** 2019-07-03

**Authors:** Evgenii Belykh, Eric J. Miller, Alessandro Carotenuto, Arpan A. Patel, Claudio Cavallo, Nikolay L. Martirosyan, Debbie R. Healey, Vadim A. Byvaltsev, Adrienne C. Scheck, Michael T. Lawton, Jennifer M. Eschbacher, Peter Nakaji, Mark C. Preul

**Affiliations:** ^1^Department of Neurosurgery, Barrow Neurological Institute, St. Joseph's Hospital and Medical Center, Phoenix, AZ, United States; ^2^Department of Neurosurgery, Irkutsk State Medical University, Irkutsk, Russia; ^3^Department of Neuro-Oncology Research, Barrow Neurological Institute, St. Joseph's Hospital and Medical Center, Phoenix, AZ, United States; ^4^Department of Neuropathology, Barrow Neurological Institute, St. Joseph's Hospital and Medical Center, Phoenix, AZ, United States

**Keywords:** 5-ALA, brain tumor, confocal, endomicroscopy, fluorescein, fluorescence, glioma, imaging

## Abstract

**Background:** Previous studies showed that confocal laser endomicroscopy (CLE) images of brain tumors acquired by a first-generation (Gen1) CLE system using fluorescein sodium (FNa) contrast yielded a diagnostic accuracy similar to frozen surgical sections and histologic analysis. We investigated performance improvements of a second-generation (Gen2) CLE system designed specifically for neurosurgical use.

**Methods:** Rodent glioma models were used for *in vivo* and rapid *ex vivo* CLE imaging. FNa and 5-aminolevulinic acid were used as contrast agents. Gen1 and Gen2 CLE images were compared to distinguish cytoarchitectural features of tumor mass and margin and surrounding and normal brain regions. We assessed imaging parameters (gain, laser power, brightness, scanning speed, imaging depth, and Z-stack [3D image acquisition]) and evaluated optimal values for better neurosurgical imaging performance with Gen2.

**Results:** Efficacy of Gen1 and Gen2 was similar in identifying normal brain tissue, vasculature, and tumor cells in masses or at margins. Gen2 had smaller field of view, but higher image resolution, and sharper, clearer images. Other advantages of the Gen2 were auto-brightness correction, user interface, image metadata handling, and image transfer. CLE imaging with FNa allowed identification of nuclear and cytoplasmic contours in tumor cells. Injection of higher dosages of FNa (20 and 40 mg/kg vs. 0.1–8 mg/kg) resulted in better image clarity and structural identification. When used with 5-aminolevulinic acid, CLE was not able to detect individual glioma cells labeled with protoporphyrin IX, but overall fluorescence intensity was higher (*p* < 0.01) than in the normal hemisphere. Gen2 Z-stack imaging allowed a unique 3D image volume presentation through the focal depth.

**Conclusion:** Compared with Gen1, advantages of Gen2 CLE included a more responsive and intuitive user interface, collection of metadata with each image, automatic Z-stack imaging, sharper images, and a sterile sheath. Shortcomings of Gen2 were a slightly slower maximal imaging speed and smaller field of view. Optimal Gen2 imaging parameters to visualize brain tumor cytoarchitecture with FNa as a fluorescent contrast were defined to aid further neurosurgical clinical *in vivo* and rapid *ex vivo* use. Further validation of the Gen2 CLE for microscopic visualization and diagnosis of brain tumors is ongoing.

## Introduction

Intraoperative diagnosis in neurosurgery has traditionally relied on frozen and formalin-fixed, paraffin-embedded section analysis of biopsied tissue samples. Although this technique is considered to be the “gold standard” for establishing a histopathologic diagnosis, it entails a number of significant limitations. These limitations include the time required for transferring, processing, and interpreting the tissue (1); the presence of artifacts and sampling errors (1–3); as well as the differences present when comparing frozen and permanent sections that may lead to misdiagnosis (1, 4). Rapid intraoperative diagnosis has become possible with refinement and miniaturization of the research-type confocal laser scanning microscope into a handheld confocal laser endomicroscopy (CLE) system (5, 6). Combined with appropriate fluorescent stains or labels, CLE provides an imaging technique for real-time intraoperative *in vivo* visualization of histopathologic features of suspected tumor and healthy tissues.

Previous studies using a CLE system originally designed for nonneurosurgical use (e.g., gastrointestinal luminal examination) showed that a blinded review of CLE imaging of brain gliomas and meningiomas by a neuropathologist yielded an accuracy rate of 92.9%, similar to those previously reported with frozen-section analysis (4, 7–9). In terms of analysis of tumor margins, CLE has not yet been thoroughly investigated in human brain tumors; however, in experimental animals, CLE was able to visualize border regions of glioma (10, 11). In humans, CLE was shown to increase accuracy of delineation of margins in early gastric cancer (12). CLE may be used as a diagnostic tool, but it also has the potential to aid in optimizing surgical resection of brain tumors, including maximal safe tumor resection, which is a strategy that would be expected to have a positive impact on long-term neurosurgical patient survival, especially among patients with invasive malignant tumors (10, 13–18).

Various CLE devices have been developed; the proximal scanning fiber optic CLE (19, 20) and distal scanning CLE (1) systems are the most advanced in terms of potential for becoming adopted into wide clinical use (21). Importantly, each CLE system has different pre-set, mostly unchangeable, imaging parameters that may result in unique and different diagnostic performance; therefore, meticulous independent assessment of each microscope system is required. We previously evaluated the diagnostic performance and other application parameters of a handheld scanning CLE system (Optiscan Pty., Ltd., Mulgrave, Australia), referred to in this report as the first-generation (Gen1) device and designed for gastrointestinal use. This system has recently served as the imaging platform for development of a second-generation (Gen2) CLE system specifically aimed at neurosurgery.

The Gen2 CLE device was designed to function specifically for intraoperative application in neurosurgery and to integrate with the robotic operating microscope visualization platform for neurosurgery (22). The goals for this device in neurosurgery are ultimately to increase the positive yield of biopsies and to serve as a tool to microscopically explore, in portable and rapid fashion, for tumor cells beyond the obvious margin of infiltrating tumors, such as cells in and around the surgical resection bed, or to define suspected tumor invasion within eloquent cortex. Previous studies regarding *in vivo* investigation of distal scanning CLE in human and animal model brain tumors were conducted using the Gen1 device, which had several performance characteristics that limited its use in neurosurgery. These included lack of a sterile attachment cover sheath for the imaging probe, a different design and function of the handheld probe that was not optimal for the neurosurgeon's usual hand position, and nonoptimal imaging processing and display (1, 7, 10, 23–25). Therefore, this study was designed to assess performance of the Gen2 CLE system, assess differences in image quality or diagnostic accuracy of the Gen1 and Gen2 systems, and provide additional information on the probe conformation, and optimal handling practices that were designed to produce high-quality confocal intraoperative images for neurosurgery on-the-fly.

Gen1 and Gen2 functionalities were compared using fluorescein sodium (FNa), the primary fluorophore with which they were designed to work, and with 5-aminolevulinic acid (5-ALA)-induced protoporphyrin IX (PpIX) fluorescence. Although the fluorescence signal from PpIX is not within the optimal detection range for these CLE systems, 5-ALA is also of interest in brain tumor surgery, especially for use in invasive gliomas, and our preliminary studies suggested that the PpIX signal may be faintly detected with the CLE systems. This study aimed to expand on the current literature related to CLE for brain tumor imaging, including further investigating the relationship between FNa dosage and image quality, the ability to differentiate cellular structures with CLE and FNa, and the utility of both generations of CLE with 5-ALA for the detection of tumor tissue.

## Methods

### Ethics Approval

All animal investigations were performed according to the guidelines outlined by the National Institutes of Health *Guide for the Care and Use of Laboratory Animals* and with approval from the Institutional Animal Care and Use Committee of the Barrow Neurological Institute and St. Joseph's Hospital and Medical Center, Phoenix, Arizona. Animals were maintained under approved veterinary care in the vivarium of St. Joseph's Hospital and Medical Center.

**Figure 1:**
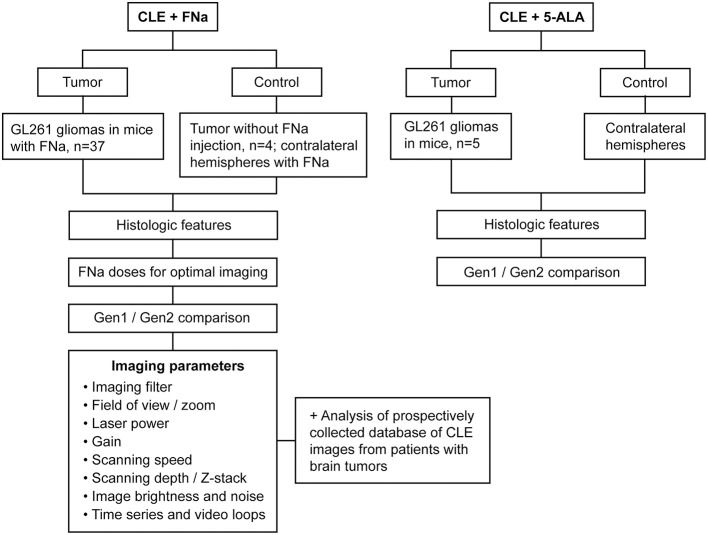
Study design flowchart. CLE, confocal laser endomicroscope; FNa, fluorescein sodium; Gen1, generation 1; Gen2, generation 2; 5-ALA, 5-aminolevulinic acid. Used with permission from Barrow Neurological Institute, Phoenix, Arizona.

Patient tissues used for this project were acquired from a prospective ongoing brain tumor clinical study. Patients with preoperative diagnoses of brain masses requiring surgical removal and for whom the decision was to use the assistance of fluorescence surgical guidance with FNa were prospectively enrolled. Extra biopsies from tumor tissue that would have to be safely removed during the normal course of the surgery were used. De-identified samples were placed on a wet telfa and submitted for immediate *ex vivo* CLE analysis. The same samples were then sent for routine histologic processing and review by a neuropathologist. All patients gave voluntary informed consent as a part of a study protocol approved by the Institutional Review Board of the Barrow Neurological Institute, St. Joseph's Hospital and Medical Center, Phoenix, Arizona. A flowchart describing the study protocol is presented in Figure 1.

### Mouse Glioma Model

Ten-week-old female B6(Cg)-Tyr^c−2J^/J mice (albino variant C57BL/6J, The Jackson Laboratory, Bar Harbor, ME) weighing 20 g (mean) were anesthetized and placed in a small-animal stereotactic head frame. Glioma models were created according to the previously described protocol (26). Briefly, 2 μL of GL261-luc tumor cells (1.45 × 10^7^ cells/mL; Division of Cancer Treatment and Diagnosis, National Cancer Institute, Bethesda, MD) were infused 2 mm lateral and 2 mm posterior to bregma at a depth of 2.5 mm from the brain surface through a bur hole at the targeted brain location after the syringe needle was withdrawn 0.5 mm to a total depth of 2 mm below the surface of the brain to create a 0.5 mm pocket for the cells. The 2-μL cell suspension was infused using a controllable microinjector (0.67 μL/min × 3 min with the needle in place for 2 min afterward) (10).

A week prior to surgery, the mice were injected with 15 μg/kg of luciferin (PerkinElmer, Waltham, MA), anesthetized using isoflurane in a 37°C chamber, and glioma growth was confirmed using bioluminescence detection in the IVIS Spectrum *in vivo* imaging system (PerkinElmer). Bioluminescence images were examined and quantified in Living Image 4.3 software (PerkinElmer) (26).

### Drug Administration

CLE imaging was performed within the first 2 h after FNa injection. We used escalating dosages of FNa: 0.1 (*n* = 4), 1 (*n* = 7), 2 (*n* = 2), 5 (*n* = 7), 8 (*n* = 5), 20 (*n* = 6), and 40 (*n* = 6) mg/kg. These doses approximate the doses of FNa that can be used in humans, as we have previously used 5 mg/kg IV in clinical study. The 5-ALA (Clinical grade, NIOPIK, Moscow, Russia) was injected intraperitoneally 2 h before surgery at a dose of 5 mg dissolved in 200 μL of normal saline (*n* = 5). Chosen dosages are similar to those previously used in comparable animal studies with 5-ALA (27–29) and FNa (10, 30–32) and are similar to or higher than those used in humans to compensate for differences in metabolism.

### Intraoperative CLE Imaging

After confirmation of tumor growth and size, on average 21 days after implantation, the mice underwent craniectomy. The animals were anesthetized, and their oxygen supply and body temperature were maintained throughout the procedure. An operating microscope (Pentero 900, Carl Zeiss AG, Oberkochen, Germany) was used to visualize the exposed surfaces of both cerebral hemispheres. Brain tumors were identified macroscopically in the right hemisphere of all mice, and the CLE scanning was performed. After imaging was performed, the anesthetized animals were euthanized according to protocol guidelines. Brains were extracted, sliced fresh coronally (1 mm in thickness) through the center of the tumor in a mouse brain slicer matrix (ZIVIC Instruments, Pittsburgh, PA), and imaged rapidly *ex vivo* with CLE again.

Gen1 (Optiscan 5.1, Carl Zeiss AG) and Gen2 CLE (Convivo, Carl Zeiss AG) were compared in terms of intraoperative fluorescence visualization using either FNa (*n* = 37), 5-ALA (*n* = 5), or no dye injection (*n* = 4 control). This comparison was completed initially using the Gen1 CLE in a designated area. Once adequate images were acquired, the Gen2 CLE was used to take further images at the same area. All scanning was performed *in vivo* and then *ex vivo* within the 2-h interval alternating Gen1 and Gen2 confocal microscopes targeting multiple regions of interest. Further testing included evaluation of Gen2 imaging with different available filters (green bandpass or green longpass and red longpass) and with various gain, laser intensity, brightness, acquisition speed, and zoom settings controlling for image quality and noise levels. In 2 animals, topical acridine orange was used for nuclear staining as described elsewhere (10).

### Operating Microscopy Fluorescence Imaging

Gross FNa and protoporphyrin IX fluorescence in the brain was assessed with the operating microscopes equipped with the Yellow 560 and Blue 400 filters, respectively (Pentero 900 and Kinevo 900, Carl Zeiss AG).

### Benchtop Confocal Laser Microscopy

The benchtop laser scanning microscopy (LSM) imaging (LSM 710 DUO, Carl Zeiss AG) was performed with a C-Apochromat 40×/1.20 W Korr M27 objective. Sliced fresh brain samples were imaged on 35-mm glass-bottom Petri dishes (MatTek, Ashland, MA). For PpIX visualization, the excitation and detection wavelengths used were 405 and 635–750 nm, respectively. Additionally, topical staining was performed with acridine orange (AO), acriflavine (AF), Hoechst 33342 (ThermoFisher Scientific, Waltham, MA), and 4′6-diamidino-2-phenylindole (DAPI). The excitation wavelength was 488 nm for AO, AF, or FNa, and 405 nm for DAPI and Hoechst; and the detection wavelength was 493–625 nm for AO, AF, or FNa, and 410–585 nm for DAPI and Hoechst. Examples of imaging modalities are shown in Supplemental Figure 1.

### Image Assessment and Statistical Analysis

Imaging analysis and cell and nuclei size measurements were performed in FIJI software (open source software) (33). Statistical analysis was completed in Excel (Microsoft, Redmond, WA). Quality of CLE imaging was scored independently by 5 respondents trained in interpreting CLE images as bad (1), average (2), good (3), or excellent (4), based on the ability to visualize characteristic GL261 tumor histologic characteristics and patterns. Grading was performed based on the assessment of the best confocal images from each animal. Student *t*-test, Mann-Whitney U test, and Kruskal-Wallis analysis of variance (ANOVA) with significance set at *p* < 0.05 were used to establish whether differences were statistically significant, and the Spearman *R*-value was used to assess correlations.

## Results

### Assessment of CLE Imaging Parameters

To produce clear images, several parameters were controlled in both systems: gain, brightness level, laser power, scanning speed, imaging depth, and Z-stack thickness (Gen2 only). Because many imaging parameters were similar between Gen1 and Gen2 CLE, salient results are presented of observations for Gen2, and are specifically mentioned when differences were observed with Gen1. Gen2 imaging parameters are summarized in Table 1.

**Table 1:** Confocal laser endomicroscopy (CLE) imaging parameters.

**Parameter**	**Gen1**	**Gen2**	**Comment**
Laser wavelength	488 nm	488 nm	Always the same
Laser power	Range: 0–1,000 μm	Range: 0–100%	Percentages correlate to the actual laser power. Fifty percent results in ~500 μm laser power; 10%, 100 μm. TIFF image metadata records actual laser power in microwatts, which may be used for further analysis
Depth	Displayed only relative value showing the arrow position on the horizontal scale; not recorded	Range: −50 to 200 μm; also displayed as relative value on the vertical scale	Knowledge of depth position is crucial for understanding of imaging location and adjustment of depth on the fly for optimal image quality. Awareness of depth helps to avoid low-quality images by not imaging too deep into a tissue. The axial imaging resolution is <4.5 μm
Brightness	Range: 0–100%	Range: 0–100%	The brightness is the built-in adjustable proprietary setting parameter used during CLE imaging. Unlike the gain and laser power, the brightness could be adjusted during the imaging. Knowledge of brightness is important to assess the true brightness of the fluorescent staining
Gain	Options: low, normal, high, max	Range: 1,800–3,000	Gain is an important parameter to consider, as it influences the image brightness. We mostly used the system with gain at the midlevel (normal or 2,400). We found it useful to increase or reduce the gain for imaging of very bright and dark fluorescent samples respectively
Speed (resolution)	Options: 1024 × 1024 μm 475 × 475 μm	Options: 1,080 540 270 135	Numbers represent the number of horizontal lines that the laser travels during image acquisition. Acquisition speed 1,080 results in a 1,920 × 1,080 pixel scan at <0.5 μm axial resolution; speed 540 results in a 1,920 × 540 pixel scan, etc. Scan area–aspect ratio is kept the same as 16 × 9. Most images are taken in the “high-image quality low-speed” mode (1,080). Higher speeds (270 and 135) are rarely used and do not produce diagnostic-quality images. Higher speed might be adjusted for an initial search of an optimal imaging position to quickly verify the presence of fluorescence. At the settings window, the user can select two speeds that would be rapidly available to choose from on the primary screen
Zoom	Constant; changes not available	Options: 1× 1.4× 2×	Zoom is usually set at 1× giving a FOV size of 475 × 267 μm (width/height). This FOV size is 2× less compared to Gen1 system; however, with FNa Gen2 provides subjectively similar appearing images to Gen1. We almost never use 2× zoom with FNa, as we feel that a larger field of view is favorable for imaging with FNa. However, 1.4× and 2× zoom are useful with other fluorophores like acridine orange or acriflavine, which bind to the cellular structures and therefore result in more contrasted images
Filter	Options: Green fluoro (505–585 nm) Green-red fluoro (505–750 nm)	Options: Gray filter Green bandpass Green longpass Red filter	Our experience with FNa is that the green longpass filter provides overall brighter images than the green bandpass filter. The red bandpass filter may be unnecessary for the work with FNa. We were not able to detect reliable PpIX (peak emission~630 nm) signal with red or green longpass filters, which we believe is mostly due to the absence of an appropriate excitation laser source (405 nm for PpIX)
Z-stack	Not available	Start position offset, range: −50 to +47 μm End position offset, range: −47 to +50 μm Step size, range: 3–20 μm	We find that for most cases +10 μm and −15 μm offsets (total depth 25 μm) with the minimally possible (3 μm) Z-step are the most optimal for 3-dimensional reconstruction of the Z-stack
Time stamp	Not available on the image	2017-04-14_12-39-20-34	Time stamp is critical for matching individual CLE images with the time on microscope and navigation in order to track the optical biopsy location

*FOV, field of view; FNa, fluorescein sodium; Gen1, first generation; Gen2, second generation; PpIX, protoporphyrin; TIFF, tagged image file format*.

#### Field of View

The field of view (FOV) of the Gen2 (475 × 267 μm, 1980 pixels/line) was half the size of the Gen1 image (475 × 475 μm, 1080 pixels/line). Gen2 images were essentially twice the resolution. Gen2 images appear sharper, but upon magnification, they have a grainier appearance compared with Gen1 images. This graininess is due to the raw image TIFF format of Gen2 images, compared to compressed and smoothing JPG formatting of Gen1 images.

#### Gain

Specimens with grossly average fluorescence brightness produced clear CLE images on Gen2 gain setting of 2,400 (Table 1). When the brain tumor appeared grossly intensely bright yellow, lowering the gain allowed imaging with a CLE brightness level within a 20–60% range, which produced optimal quality images without oversaturated pixels and with low noise. However, in specimens with low gross fluorescence intensity, the quality and resolution of CLE images did not improve regardless of the changes in imaging parameters. The increased level of black noise with an increase of brightness level and laser power only lowered image quality in dark images.

#### Scanning Speed

Scanning speed was critically important for time series imaging. Comparison of imaging speed recorded for both generations of CLEs is presented in Supplemental Table 1. Scanning speed of Gen1 was 0.8 s per frame for a 1,024 **×** 512-pixel image and 1.2 s per frame for a 1,024 **×** 1,024-pixel image. The scanning speed for Gen2 ranged from 1.3 s per frame for the highest quality imaging to 0.27 s per frame for the lowest quality imaging. Despite the difference in scanning speed, all images were recorded and stored as 1,920 **×** 1,080-pixel red, green, blue (RGB) TIFF files.

#### Scanning Depth

Optimal imaging depth with FNa was within the first few microns in depth from the surface of the imaged tissue. The CLE Z-stack function (Gen2 only) resulted in a series of images, the first of which was a central image from the current position on the Z axis. A series of images were acquired starting from the surface and ending at the deepest position, with the Z-step image thickness selection to be between 3 and 20 μm. The interval of the scanning depth could be chosen by selecting two offset positions. The maximal range was obtained at the offsets from −47 to +50, which resulted in a 97-μm thick Z-stack. The number of images per Z-stack was limited by the size of the Z-step (3 μm minimum), which for the 97-μm Z-stack resulted in 35 images. Higher ranges are not practically necessary, so the available CLE Z-stack range is sufficient for use in human brain tissue. Previous experiments have shown that the maximal depth of brain tumor tissue (using FNa) for interrogation through Z-stack imaging is 36 μm in animal and 28 μm in human brain tissue (34).

Probe movements during image capturing produced similar artifacts in both Gen1 and Gen2 CLEs. Although free-hand probe positioning yielded informative images, remaining in a steady position for an optimal Z-stack was difficult for image series acquisition. A semiflexible surgical probe holder was used to decrease movement artifacts.

#### Time Series and Video Loops

**Figure 2:**
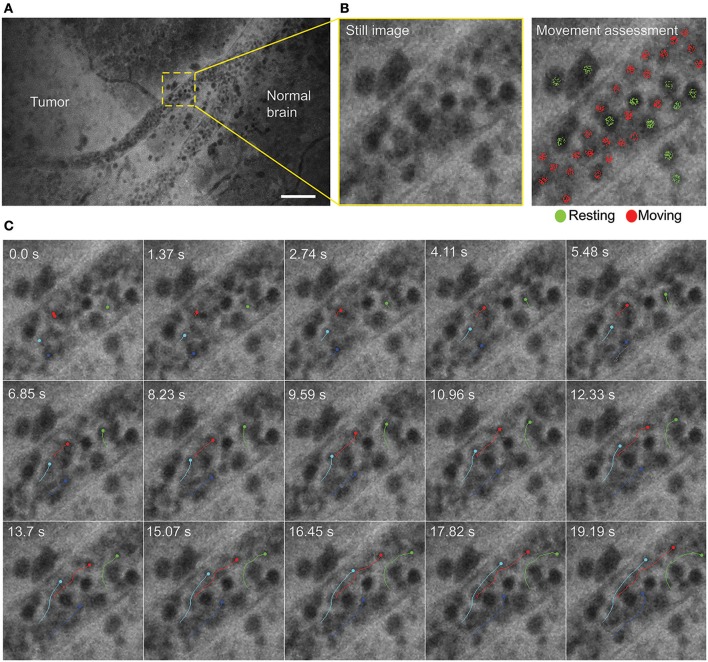
Time series imaging to differentiate stationary and mobile cells using a Gen2 confocal laser endomicroscope. **(A)** Single frame from time-lapse image. **(B)** Enlarged view of the vessel (left) and same image with moving cells labeled red and stationary cells labeled green (right). **(C)** Tracking of the selected cells shows intravascular movement of individual blood cells. Particle tracking performed in FIJI. Scale bar is 50 μm. Used with permission from Barrow Neurological Institute, Phoenix, Arizona.

Time series imaging (in both Gen1 and Gen2) allowed observation of blood cell movements inside and outside the vessels (Figure 2). Tumor cell movements seen with tissue squeezing under the CLE lens were also noted during *in vivo* imaging, which is not possible on fixed tissues. In contrast to the static hematoxylin and eosin (H&E) slide, *in vivo* CLE revealed histologic features of the living tumor cells and their behavior. In animal gliomas, many tumor cells were actually moving independently, squeezing and pushing each other, captivated by the stream of oozing blood. Such tumor cells were flowing independent of the tumor core, especially when they were at the border of the brain slice. When imaged *in vivo*, GL261 glioma cells were actually not tightly connected to one another, as they appear on H&E slides, although they grow as relatively solid tumors. CLE imaging revealed pericellular spaces that were filled with FNa, which were not previously visualized well on H&E stained slides.

### Histologic Features During CLE Imaging With FNa (Gen1 and Gen2)

**Figure 3:**
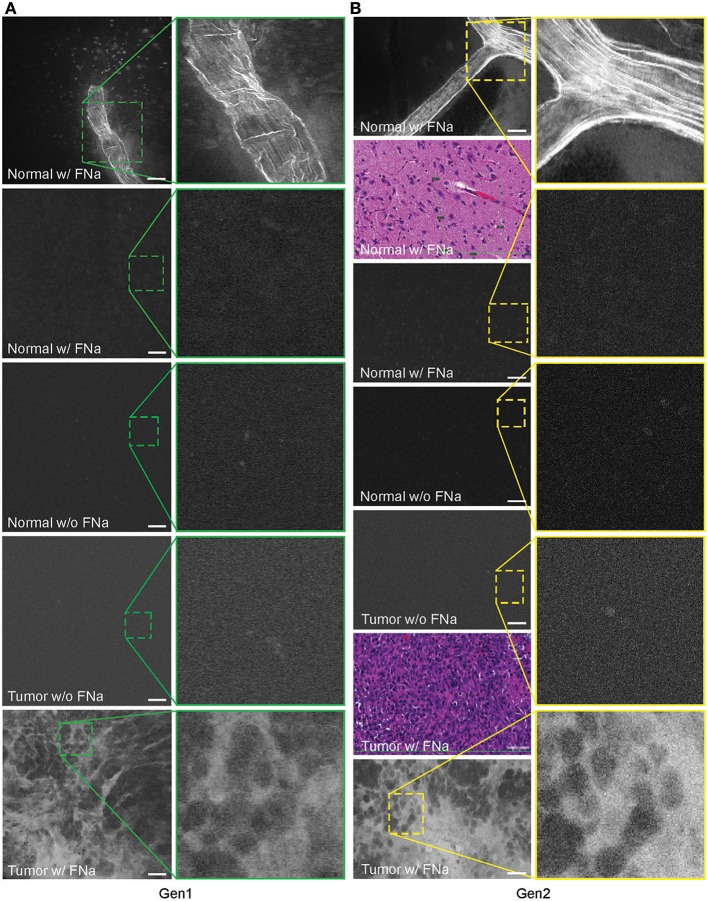
Confocal laser endomicroscopy (CLE) images of normal mouse brain, GL261 brain glioma, visualized with and without fluorescein sodium (FNa), using **(A)** generation 1 (Gen1) and **(B)** generation 2 (Gen2) CLE. Brain vasculature, brain parenchyma, and tumor staining patterns were observed with similar quality between Gen1 and Gen2 systems. The insets show similarly magnified views from Gen1 and Gen2 systems. Additional hematoxylin and eosin (H&E) images are provided from the regions of interest of normal brain and glioma areas scanned with Gen1 and Gen2 CLE. Scale bars are 50 μm. Used with permission from Barrow Neurological Institute, Phoenix, Arizona.

In no case was the tumor location uncertain based on CLE images after successful intravenous FNa injection. When imaging normal brain without tumor using CLE, FNa could be seen inside blood vessels, and occasionally leaking from vessels because of damage from surgery. Red blood cells could be visualized in these vessels as dark moving objects. No FNa was seen in other areas of the normal brain, other than intravascularly, except for sparse autofluorescent cells. Ultimately, the area of the brain away from the vessels appeared similar to the brain when FNa had not been injected, with both generally lacking fluorescence, except for rare autofluorescent cells and with limited fluorescence (Figure 3). After FNa injection, tumor cells were seen as nonfluorescent cells on a highlighted fluorescent background.

Abnormal tumor vasculature was seen in several tumor biopsy spots (Supplemental Figure 2). A gradient of FNa in the tumor was observed on images and may suggest proximity to the tumor vessels (Supplemental Figure 3).

**Figure 4:**
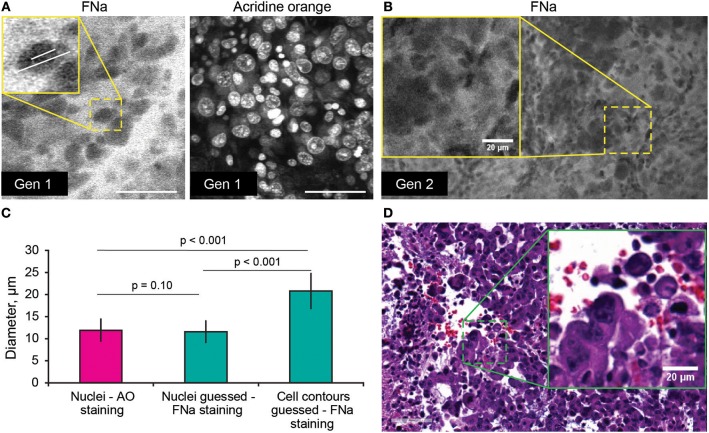
**(A,B)** Comparison of the tumor cellular features visible on confocal laser endomicroscopy images with fluorescein sodium (FNa) and acridine orange (AO). AO staining of the same specimen shows true nuclei size. Gradient of FNa distribution delineates cell contours with bright gray, while cell nuclei may be visible in some of the cells in a darker gray. **(C)** Comparison of the nuclei size measured on the images with AO staining to the nuclei size measured on the images with the FNa staining (*n* = 106 cells measured with AO stain; *n* = 53 nuclei and *n* = 52 cell diameters were measured with FNa stain). The average nucleus size determined on the FNa images was similar to the true average nucleus size based on the AO staining. Generation (Gen) 2 CLE image **(B)** shows anisocytosis, similar to **(D)** histologic findings. Scale bar in **(A)** is 50 μm. Used with permission from Barrow Neurological Institute, Phoenix, Arizona.

**Figure 5:**
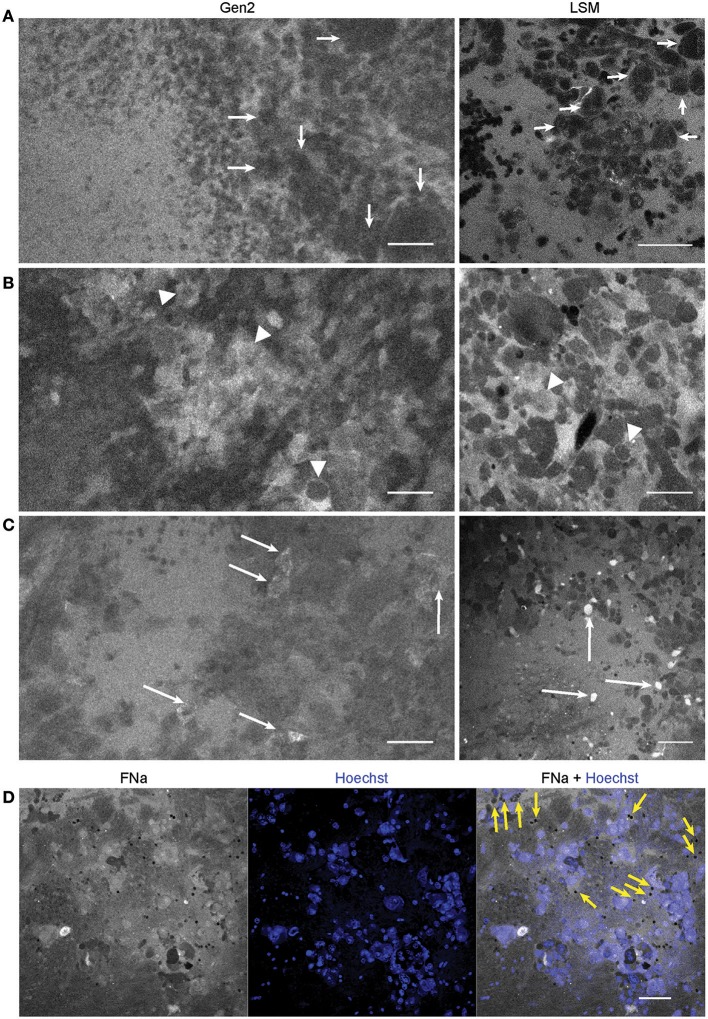
Confocal laser endomicroscopy (CLE) patterns of GL261 glioma core. *In vivo* Gen2 CLE and rapid *ex vivo* laser scanning microscopy (LSM) images show higher fluorescein sodium (FNa) signal in the tumor area and visible contours of the tumor cells. Overall tissue architecture presents similarly in CLE and LSM images. **(A)** Most often tumor cells appeared darker than the background (arrows), while in some areas **(B,C)** tumor cells absorbed FNa and appeared bright. **(D)** Red blood cells, which were not stained by the Hoechst dye, are visible on a bright FNa background (yellow arrows). Scale bars are 50 μm. Used with permission from Barrow Neurological Institute, Phoenix, Arizona.

Average nuclei sizes measured on AO-stained tumor images (11.9 ± 2.5 μm) were not different (*p* = 0.10) from those measured on FNa-contrasted CLE brain tumor images (11.6 ± 2.5 μm), suggesting validity of identification of nuclei on the FNa-based confocal images. The average nuclei size was significantly smaller (*p* < 0.01) than the cell sizes (20.8 ± 3.9 μm) (Figure 4). Benchtop confocal LSM of fresh samples stained with FNa (*in vivo*, intravenous) and Hoechst (rapid *ex vivo*, topical live cell nuclear stain) showed images similar to CLE grayscale images and provided additional confirmation regarding the dimensions of nuclei and cells observed with CLE (Figure 5).

**Figure 6:**
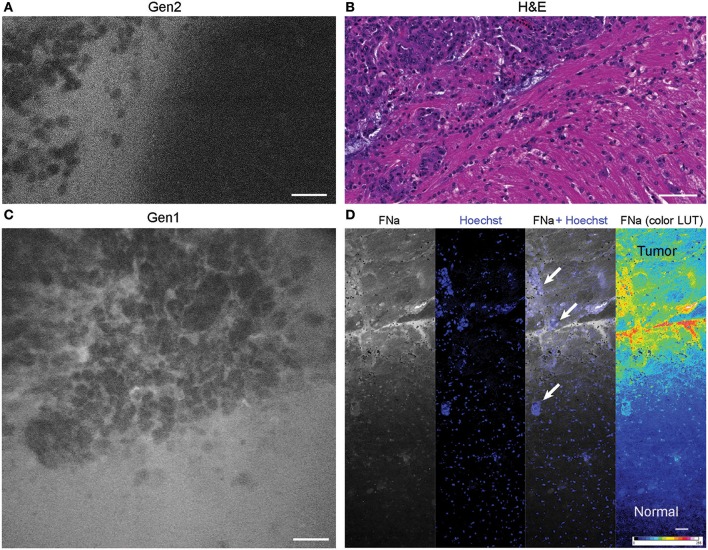
Tumor border 1 h after intravenous fluorescein sodium (FNa) injection. **(A)** Generation 2 (Gen2) and **(C)** generation 1 (Gen1) confocal laser endomicroscopy images show a similar cellular architecture pattern of the GL261 glioma border region. **(B)** Hematoxylin and eosin (H&E) image of the tumor border from a matched sample. **(D)** Larger field of view *ex vivo* benchtop confocal image shows gradient of fluorescein diffusion from the tumor to the normal brain. Arrows indicate invading tumor cell groups. LUT, look-up table. Scale bars are 50 μm. Used with permission from Barrow Neurological Institute, Phoenix, Arizona.

The tumor margin was visible in the GL261 model with both CLEs. The tumor border region appeared as a nonuniform delineation between the area with low FNa signal that did not contain atypical cellular features (normal brain) and an area with high FNa signal containing silhouettes of the abnormal tumor cells (Figure 6). Benchtop confocal microscopy of samples stained with FNa *in vivo* and rapidly counterstained *ex vivo* with Hoechst suggested that FNa signal “followed” invasions of the tumor cells in the normal brain. It confirmed that many small cell contours visible with FNa are actually anuclear red blood cells. A gradient of FNa distribution from the tumor into the normal brain was also visible with the higher FOV confocal microscopy.

When normal brain tissue was injured, leaking FNa signal was visible. Brain regions of injury without tumor could be differentiated from regions of brain with tumor on CLE images by small and nonvariable red blood cells being present, rather than large, numerous, and variable tumor cells (32).

### FNa Doses for Optimal CLE Imaging

**Figure 7:**
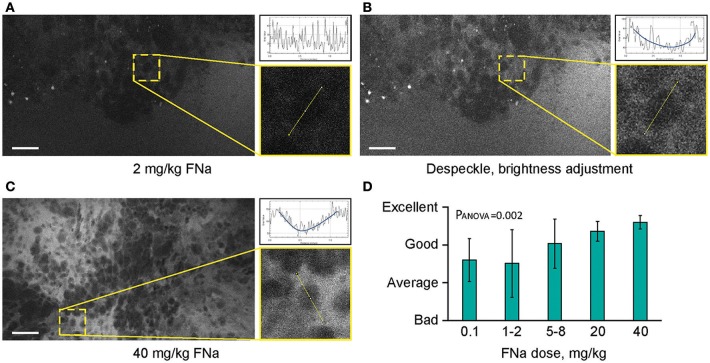
Higher concentrations of fluorescein sodium (FNa) produces images with less noise on a generation 2 confocal laser endomicroscope. Images acquired with a low dose of FNa **(A)** could be post-processed to improve brightness **(B)**, so they appear similar to higher FNa dose images **(C)**. Comparison of the enlarged regions in **(A–C)** shows that cell contours are distinguished on all images; however, noise level [diagrams in **(A–C)**] is less with a higher FNa concentration. **(D)** Subjective grading of overall CLE imaging quality is presented as a mean of all grades from 5 independent raters. The image quality is significantly better with higher FNa dosages (P_ANOVA_ = 0.002). Scale bars are 50 μm. Used with permission from Barrow Neurological Institute, Phoenix, Arizona.

Different concentrations of intravenous FNa as a contrast for CLE imaging yielded variations in image appearance. Bolus injections of higher concentrations of FNa resulted in brighter images of tumors with less noise, and an overall increase in the fraction of diagnostic frames, compared to lower FNa concentrations (*R* = 0.5, *p* < 0.05) (Figure 7). The best working dosages were 20 and 40 mg/kg and are in accordance with previous publications [100 mg/kg (11); 8 mg/kg (24); 7.7 mg/kg (30)]. Based on our experience, imaging closer to the time of FNa injection produced images with greater contrast and overall higher quality compared with images obtained longer after the injection.

### Histologic Features During CLE Imaging With 5-ALA (Gen1 and Gen2)

**Figure 8:**
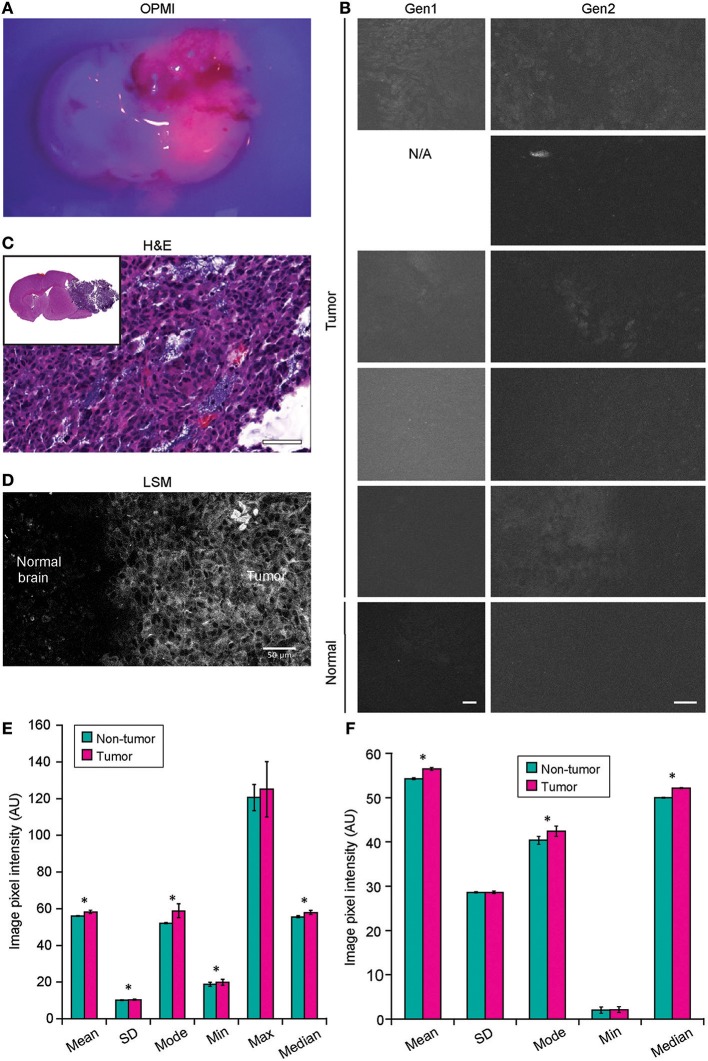
Confocal laser endomicroscopy (CLE) imaging of GL261 glioma with 5-aminolevulenic acid (5-ALA). **(A)** Representative image of a coronal brain slice with a tumor viewed through the Pentero 900 operative microscope in Blue 400 mode shows bright red fluorescence from the tumor. **(B)** The best representative generation 1 (Gen1) and generation 2 (Gen2) CLE images of tumor (rows are 5 separate animals) and normal brain 2 h after intraperitoneal 5-ALA administration. Normal brain shows no fluorescent signal in any of the cases. Fluorescent signal from tumor is very low, visible from only a few areas, and is not consistent across the biopsy locations and across the animals imaged. **(C)** Hematoxylin and eosin (H&E) coronal slice of the brain with a tumor shows hypercellular tumor in the areas from which CLE imaging was performed. **(D)** Laser scanning microscopy (LSM) image at the tumor margin shows the protoporphyrin signal localized to tumor cell cytoplasm. The image displayed in gray scale and size is similar to Gen2 CLE. (Image acquired with 40× 1.2W objective; 405 nm excitation; 598–740 nm detection range.) **(E)** Gen1 CLE imaging of GL261 gliomas and contralateral normal brain as a control with 5-ALA. Quantification of the images (*n* = 54 control; *n* = 59 tumor images from 5 animals) showed minimal but significant difference in the overall pixel intensities of the images taken from the tumor vs. normal brain. Groups were compared using Student *t*-tests; an asterisk indicates *p* < 0.01. **(F)** Quantification of the Gen2 CLE images (*n* = 5 control; *n* = 4 tumor) showed minimal but significant differences in the overall pixel intensities of the selected best images taken from the tumor and normal brain. Groups were compared using Student *t*-tests; an asterisk indicates *p* < 0.01. Selected images were acquired with similar CLE settings. Scale bars are 50 μm. AU, arbitrary unit. Used with permission from Barrow Neurological Institute, Phoenix, Arizona.

Neither Gen1 nor Gen2 CLE was reliably able to detect PpIX fluorescence in experimental tumors after 5-ALA administration. At the same time, Pentero 900 imaging with a dedicated Blue 400 filter showed very bright red PpIX fluorescence with all tumor masses. Gen2 CLE detected some signal in several tumor areas; however, this signal was not consistent in different locations imaged over the tumor area. Most imaging of the tumor showed dark images without visible signal. In all 5 animals interrogated with both Gen1 and Gen2, reliable histologic features of tumor or normal brain were not discernible with PpIX fluorescence. However, sparse fluorescent spots were observed in normal brain and tumor in experimental animals and were likely autofluorescence (35). When the overall image intensities of normal brain and tumor areas, acquired with similar parameters, were compared, they differed significantly (*p* < 0.01) (Figure 8).

### Influence of Imaging Parameters on the Image Quality (Gen2 System)

#### Imaging Filter

**Figure 9:**
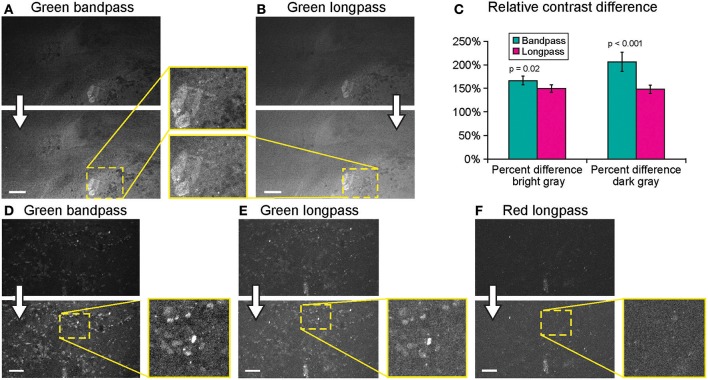
Generation 2 confocal laser endomicroscope (CLE) imaging with various filters. Comparison of the CLE images taken from the same regions of interest with green bandpass **(A,D)**, green longpass **(B,E)**, and red **(F)** optical filters. Human glioma samples (patients were injected with FNa intraoperatively) were used for analysis and illustration. Thick arrows signify post-processing in FIJI, which includes despeckling and maximum brightness adjustment. **(C)** Matching regions of interest (ROI) were selected in FIJI to compare contrast between various structures in longpass and bandpass filters. ROIs were manually drawn over the brightest structures (*n* = 5), over the surrounding gray background (*n* = 3), and on the dark round structures (*n* = 5). Paired Student *t*-tests were used for comparison. **(D–F)** Green bandpass **(D)**, green longpass **(E)**, and red longpass **(F)** filter images obtained at the same location. Scale bars are 50 μm. Used with permission from Barrow Neurological Institute, Phoenix, Arizona.

Imaging using green bandpass or green longpass filters was compared on both systems to visualize FNa in mouse glioma models. Images acquired with the longpass filter appeared brighter. At the same time, quantitative analysis showed that the bandpass filter resulted in significantly better-contrasted images (Figure 9). With autobrightness function on, some Gen2 images (using FNa or AO as a contrast) appeared subjectively dark. However, adjusting the brightness during post-processing resulted in significantly better-contrasted images than when using the longpass filter. The red longpass filter was not useful for FNa imaging; however, scant cells and structures emitted light in the red spectrum within normal brain and tumor areas.

#### Detector Gain

**Figure 10:**
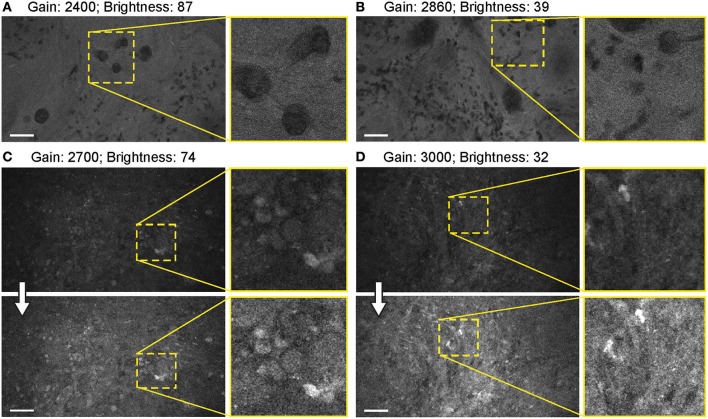
Balanced generation 2 (Gen2) confocal laser endomicroscope (CLE) images with different gain results in comparable image quality. **(A–D)** Comparison of the CLE images taken with various gain setup. Human meningioma **(A,B)** and glioblastoma **(C,D)** samples scanned *ex vivo* 2 h after fluorescein sodium (2-mg/kg) injection are used for analysis and illustration. Increase in the gain requires reciprocal adjustments in the brightness. Overall, the resulting images have comparable quality. **(A,B)** are visualized from one patient, while **(C,D)** are visualized from a different patient. Thick arrows signify post-processing in FIJI, which includes despeckling and maximum brightness adjustment. Scale bars are 50 μm. Used with permission from Barrow Neurological Institute, Phoenix, Arizona.

When FNa was used, images were acquired mainly with a gain at the mid-position (2,400). Very bright fluorescent specimens (10–40 mg/kg FNa) required gain adjustments on only rare occasions, as control was usually possible for oversaturated pixels and image quality by lowering the laser power and brightness (Figure 10).

#### Laser Power

**Figure 11:**
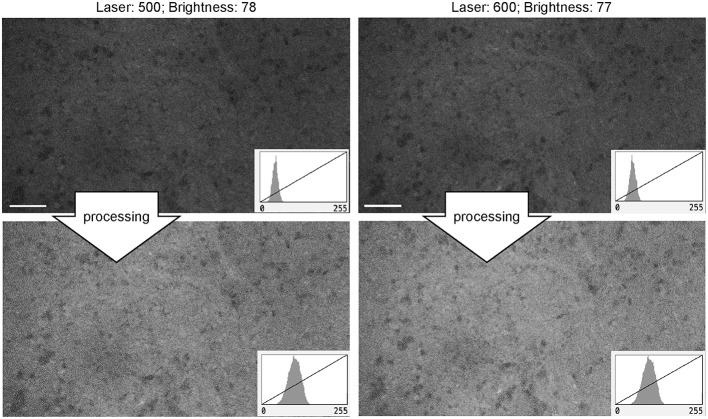
Generation 2 confocal laser endomicroscope images taken at different laser intensity values. A human meningioma sample is used for analysis and illustration. Brightness was kept at a similar level during image acquisition. Increase in the laser intensity from 500 μm (upper left) to 600 μm (upper right) leads to a brighter overall image. Further increase in laser power does not improve contrast. Thick arrows signify post-processing in FIJI, which includes despeckling and maximum brightness adjustment. There are no subjective differences in images after post-processing (lower row). Scale bars are 50 μm. Used with permission from Barrow Neurological Institute, Phoenix, Arizona.

For FNa imaging, the laser power was usually at 50% (500 μm). In locations that produced dark images (low FNa or bleached areas), increasing the laser intensity improved image quality and brightness to some extent, but further increases in laser power did not result in quality improvement. The functioning of laser power control was not different between Gen1 and Gen2 systems. Overall, grossly bright fluorescent samples resulted in excellent contrast and quality at 50% laser power and gain position in the midpoint range (2,400) (Figure 11).

#### Image Brightness and Noise

The autobrightness function auto-adjusts the brightness of the image on the fly during continuous imaging based on the brightness histogram of the precedent image, independently from the gain and laser power settings. With one exception, the autobrightness function of the Gen2 was advantageous for rapid image and optimal brightness display, contrasting with the Gen1 that lacked this function and required frequent manual adjustments. The exception occurred when the image had abnormally bright artifacts. In such situations, the autobrightness function decreased the overall image brightness, with the FNa signal in the tumor interstitium becoming low and barely detectable—this was observed both qualitatively in real time and quantitatively during post-processing. In such situations, manual brightness setup was used to obtain better overall image quality.

**Figure 12:**
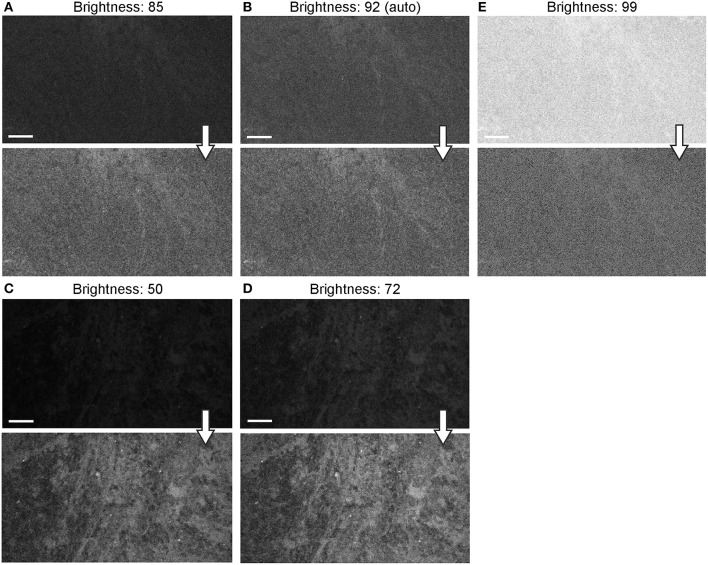
Generation 2 confocal laser endomicroscope (CLE) images taken with different brightness setup. **(A–E)** Comparison of the CLE images taken with various brightness settings. The same fields of view of meningioma **(A–C)** and low-grade glioma **(D,E)** human samples are used for analysis and illustration. Brightness is in the automatic position for **(B)**; in all other figures, brightness is set manually. Increase in brightness higher than automatic position does not increase image quality quantitatively **(C)**. Thick arrows signify post-processing in FIJI, which includes despeckling and maximum brightness adjustment, except for **(C)** in which brightness is decreased with post-processing. Scale bars are 50 μm. Used with permission from Barrow Neurological Institute, Phoenix, Arizona.

When the autobrightness function was on, the increase in gain resulted in a proportional decrease in automatic brightness level. Brightness values can be indicative of noise level. Brightness levels below 80% were the most optimal with less black noise, compared to a brightness level of 80% and above. The brightness values were maintained below 80% to keep the noise levels low (Figure 12).

**Figure 13:**
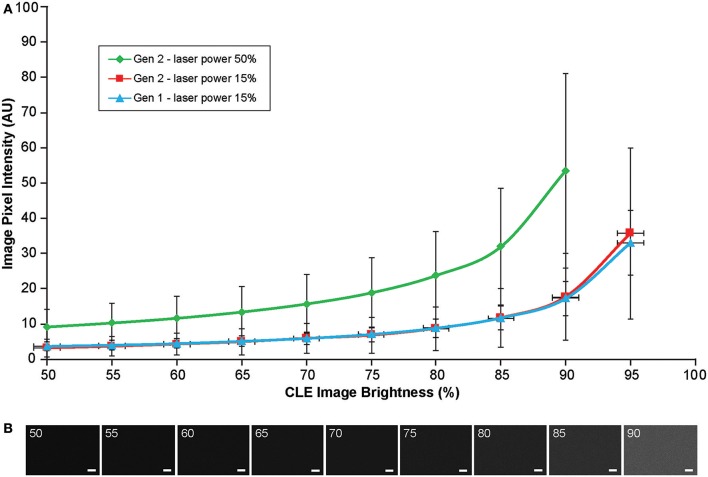
Image brightness and background noise level with the generation 1 (Gen1) and generation 2 (Gen2) confocal laser endomicroscope (CLE). Image brightness and background noise level depend on the laser power and image brightness set up of the CLE. **(A)** Graph showing observed image intensity in arbitrary units (AU) (y axis) at the various brightness positions, while imaging normal brain area. This value represents “black” noise. Brightness setting above 80% results in a significant increase of black noise, which may decrease image quality. Therefore, when imaging, one should aim at getting a picture at a brightness level below 80%. **(B)** Unprocessed Gen 2 CLE images taken at various indicated brightness levels (%) that are set manually. Scale bars are 50 μm. Used with permission from Barrow Neurological Institute, Phoenix, Arizona.

Background noise levels were further studied to assess the effects of Gen2 imaging parameters on image quality. Imaging of normal brain with various manually set brightness levels revealed that mean image intensity and standard deviation of the individual pixels had a positive correlation with brightness level (Figure 13). At a brightness of 91%, laser power of 50% (505 μm), speed of 1080 pixels (~1.26 seconds per frame), and gain of 2400, the average pixel intensity from a normal brain (black color) was 53.5 arbitrary units, corresponding to about 20% saturation of the 8-bit image.

#### Image Acquisition Speed

**Figure 14:**
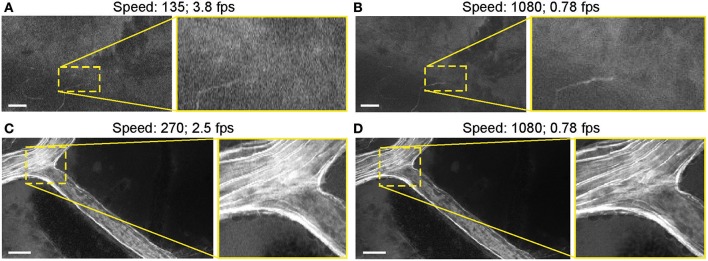
Generation 2 confocal laser endomicroscope (CLE) images taken at different speeds. Comparison of CLE images taken with 3 various acquisition speed settings: **(A)** 135, **(B,D)** 1,080, and **(C)** 270. Images acquired at an intermediate speed setting **(C)** of 270 (calculated mean 2.5 frames per second) are of average quality and are able to resolve more structural details than images taken at the faster speed of 135 **(A)**. Significant improvement in image quality at the slower speed of 1,080 **(B,D)** is mostly apparent in the areas with high contrast, such as small vessels when fluorescein sodium is used. When viewed as smaller pictures, the differences are less pronounced. However, on a large high-definition display, the image quality at different speeds differs substantially. Scale bars are 50 μm. Used with permission from Barrow Neurological Institute, Phoenix, Arizona.

Most images were acquired at a speed setting of 1080 pixels (~1.26 seconds per frame), which resulted in the best image quality (Table 1). Faster scanning at speeds of 135 pixels (~0.26 seconds per frame) and 270 pixels (~0.4 seconds per frame) were useful for rapid scanning of large areas to determine the presence or absence of an FNa signal or for blood flow imaging (Figure 14). Some gross tumor tissue structures were visible at speeds of 135 and 270 but appeared overly pixelated on the CLE display. Image quality and contrast were significantly better at 1080 pixels (~1.26 seconds per frame), especially in areas with small vessels contrasted with FNa or when using nuclear stains (AO, AF).

## Discussion

The Gen2 CLE system is compatible with the specific demands of neurosurgical use. With the disposable sterile sheath that includes a coverslip, the Gen2 CLE can now be safely used on the human brain during surgery without the need for a complex sterilization procedure after surgery, as was the case with the Gen1 system (Supplemental Figure 4). The ergonomics of the probe conformation are such that it resembles a curved, lightweight neurosurgical suction device that will lie comfortably in the hand, grasped by the first few fingers, in a fashion that allows smooth, stabilized movement as a neurosurgeon is used to for surgical instruments. In this study, we performed a preclinical investigation of the Gen2 CLE and characterized its operation, capabilities, and limitations, and compared its performance to the Gen1 CLE system.

### Image Quality Comparison (Gen1 vs. Gen2)

#### Imaging Parameters (Gen1 and Gen2)

Multiple imaging parameters can be adjusted in both Gen1 and Gen2 systems making them flexible and capable of detecting a single-channel fluorescent signal over a broad range of intensity values. Gen2 is more adjustable, as it allows for changing most parameters rapidly during scanning, while with the Gen1, the user is required to create a new imaging session using the operating software interface to change the gain or filter. Another convenient advantage is that, unlike the Gen1, the Gen2 imaging parameters are recorded as part of the file properties. Having access to these parameters is important for quantitative analysis and comparison among various images, especially to evaluate fluorophore signal intensity in the image, which correlates with the concentration of the fluorophore in the sample. For FNa-guided optical biopsy in brain tumors, such information may be used to assess the degree of blood-brain barrier disruption, to eliminate imaging artifacts, to identify potential causes of suboptimal images, or to improve images in post-processing.

#### Still Images (Gen1 and Gen2)

While continuous imaging occurs at a chosen speed in both CLE systems, the recording options are different. Both systems have the option to record a single still image and time series, while the Gen2 can also automatically record Z-stacks. Still image recordings were used when the sample quality was perfect and when there was no concern about having only a few selected imaging frames recorded while interrogating the tissue. However, in most cases, continuous recording was preferred and advantageous, which is discussed later. Finding the exact same position again is nearly impossible with a CLE system due to the minute dimensions on the order of fractions of a millimeter (475 × 475 μm for Gen1; 475 × 267 μm for Gen2), contact probe design, and pliable nature of unfixed brain tissue. Thus, there was always a concern about not being able to return the CLE to the previous imaging plane. Therefore, still recordings were used less frequently than Z-stacks or time series. Incorporation and registration of the probe into an image-guided surgery navigation system would allow improvement in reassessing tissue imaging location.

#### Z-Stack Function (Gen2 Only)

A Z-stack is a series of images taken in rapid succession at different depths and a constant position (34). The “Z-step” is the depth distance between images and is a novel feature for handheld CLE imaging with the Gen2 CLE system. The range of the Z-stack can be set by the user, and the Z-step is a constant 3 μm, resulting in 2–35 images per stack. At least several slices (maximum 10–13) from the Z-stack were acquired at optimal Z positions that were in focus, usable, and diagnostic (36). Informative images from a Z-stack can be easily processed into video loops or semitransparent volumetric images for detailed assessment and further interpretation.

It is also worth mentioning that the size of objects becomes larger when they are imaged out of focus and deeper within the tissue. This could potentially make small red blood cells look larger than they are, thus imposing a minor potential for misdiagnosing small cells as tumor cells.

#### Time Series Recording and Video Loops (Gen1 and Gen2)

Time series (or “cine”-series) recordings were used extensively for post-processing and creation of video loops. Continuous nonstop recording during Gen1 or Gen2 CLE interrogation of the sample was helpful compared with “on-demand” snapshot recording because with the latter technique, some locations were bleached out before the image was recorded. Gradual photobleaching was observed, but with practice, minimizing the scan time avoided this problem. We did not investigate whether laser interrogation results in tissue damage. Although tissue damage is unlikely, we are conducting an ongoing investigation of the potential phototoxicity of CLE scanning. Additionally, minute tissue shifts changed the observed image before taking a picture, and the probe often could not be returned to the desired location because of the pliable nature of unfixed brain tissue. Z-position, brightness, and laser power can be adjusted during the time series imaging to record optimal quality images.

Notably, time series imaging revealed a “movement” feature of the tissue and supplemented visual information gained from still images. This feature is useful in several aspects for tissue histologic characteristics and CLE image interpretation. Primarily, differentiation of freely moving red blood cells from other moving or stationary cells becomes much easier and thus fosters the diagnostic accuracy of image and tissue interpretation. Additionally, it becomes easier to differentiate real signal from noise. By viewing time series, differentiation is possible between what is meaningful and what is merely stochastic noise. Such judgment is usually difficult on still images because of the nature of FNa. FNa is a nonspecific dye that is not bound to any structure, but rather provides a bright background (i.e., a silhouetted appearance) on which to observe, contrast, and differentiate structures. Noise is an inherent limitation of FNa. *In vivo* CLE demonstrated dynamics of the glioma environment for the first time, something that is not possible in fixed brain tissue.

It should also be noted that CLE allowed for *in vivo* tracking of blood flow in vessels. Tracking of individual blood cells is not always possible because of fast movements that require high-speed scanning confocal imaging systems, such as spinning disk confocal microscopes (37–39).

#### Histologic Pictures Visualized by CLE With FNa (Gen1 and Gen2)

The use of FNa with both distal scanning CLE systems was effective to visualize brain vasculature, red blood cells, silhouettes of neoplastic cells, nuclear and cell dimensions, GL261 glioma border, and brain injury. FNa aided detection of normal and tumor vessels and did not significantly highlight normal brain tissue. Cell nuclei and cytoplasm may be distinguished in some of the cells by a gradient of FNa penetration through different biological membranes (Supplemental Figure 5). FNa extravasation due to injury can be differentiated from the FNa diffusion in the tumor using characteristic histologic patterns on CLE (32). It is worth noting that individual fluorescent cells were frequently observed in both tumor and normal brain (Supplemental Figure 6). This phenomenon had various patterns and was observed more frequently with increased imaging time after FNa injection. Singular and multiple cells were observed that took up FNa on the cut surface of the tumors. Even though such images did not represent a characteristic CLE image of the tumor, they were helpful to assess the cell morphology, tissue architecture, and cytoplasm-to-nucleus ratio. The reason for such FNa uptake by some cells is still unknown because tumor cells do not show FNa uptake *in vitro* (30). However, it might be due to cell membrane disruption caused by injury, as such findings were seen mostly in *ex vivo* samples. Additionally, large singular cells that took up FNa seen in the tumor may be macrophages.

When considering FNa imaging strategies, continuous cine-like time series image recordings and Z-stacks were the most useful and productive. Continuous recordings were used to identify red blood cells and better appreciate cell morphology based on cell movement while reformatted volume views of Z-stacks provided an improved understanding of the 3-dimensional structure of the tissue.

#### Histologic Pictures Visualized by CLE With 5-ALA (Gen1 and Gen2)

The combination of metabolic dye 5-ALA with either Gen1 or Gen2 was not a reliable method for identification of tumor in our observations, as characteristic histologic features could not be identified. The laser wavelength of both systems (488 nm) is not optimally tuned for excitation of PpIX, and CLE images did not show specific information. CLE images did not reproduce findings when LSM with the appropriate excitation laser was used to image PpIX in the same mouse glioma model (40).

While it was possible to differentiate glioma from normal brain tissue by comparing overall average image intensity, this charcteristic was not deemed useful. Similar “fluorescence intensity” sampling has been shown previously using spectrophotometric methods (41–43). Of note, sparse fluorescent “spots” seen using the Gen2 CLE with 5-ALA were consistent with the previous intraoperative observations of patients with brain tumors with the Gen1 CLE, although the exact identification of these PpIX fluorescent signals is not confirmed (25). These structures are likely autofluorescent due to lipofuscin (35). Imaging of histologic patterns created by 5-ALA-induced PpIX in brain tumors remains limited to benchtop confocal microscopes, or hand-held fluorescence microscopes specifically designed for PpIX excitation and detection, such as a dual axis confocal microscope (44) or a scanning fiber endoscope system (40).

### CLE Parameters for Best Image Quality With FNa (Gen2 Only)

#### Filter

The first parameter to consider during CLE imaging is the filter. Between the two types of green filters (i.e., the longpass filter and bandpass filter), the longpass filter produced brighter images. Although images with such longpass filters may look more pleasing, they include nonspecific light from the background that attenuates FNa-induced contrast. Ultimately, the bandpass filter produced images with higher contrast and was preferable for obtaining better quality and interpretable images.

#### Gain

Unlike in the benchtop confocal microscope, where the gain is fine-tuned during the constant image capturing, with the Gen2 CLE the gain control is located in a separate window and is not available for rapid adjustments on the fly. However, adjustments of brightness and laser power were enough to obtain quality images in most cases without the need to adjust the gain. Only in a few very bright or very dim specimens was there a need to adjust the gain for optimal image quality.

#### Laser Power

Laser power at mid-range (50% or 500 μm) was generally optimal for imaging, with increasing intensity providing some improvements in image quality. Lack of significant improvement in image quality with increasing laser power is mainly explained by the nature of FNa, which, after extravasation, is diffused through almost all tissue thickness. Therefore, increased excitation results in increased emission in all image regions, including background and cells. Moreover, autofluorescence and noise begin to be visible at higher laser settings. This association of laser power and gain with contrast and autofluorescence is shown schematically in Supplemental Figure 7. Compared with targeted fluorophores or reflectance imaging (45), imaging of FNa is always associated with higher levels of noise.

#### Brightness

The brightness is the built-in adjustable proprietary setting parameter used during CLE imaging. It has a range of settings from 0 to 100% and, unlike the gain and laser power settings, could be adjusted during the imaging. Brightness was a primary setting of both CLE systems. Autobrightness is a new Gen2 function that saved time and made imaging process faster. Images acquired in autobrightness mode had good quality, comparable with the quality of images acquired at manually set brightness levels. Additionally, an optimal brightness level, or even pseudocoloring, can be applied later during post-processing (34).

#### Speed

The speed of image acquisition was inversely correlated with image quality. This relationship illustrates the tradeoff of the convenience of using high-speed imaging to locate informative structures quickly within a large area with little detail versus taking more time to look at higher resolution images in greater detail. For areas of high contrast, such as intact vessels with FNa within the normal brain, or pronounced hypercellular tumor areas with bright FNa nearby, high-speed imaging was able to resolve structures well. However, in most cases, fast scanning was not able to resolve red blood cells and fine cellular details.

#### Zoom

Although the FOV of Gen2 images is about half the size of Gen1 images, it was sufficient to obtain similar image interpretation for corresponding histological structures. Subjective image quality appraisal between the systems is dependent on the display size. The Gen2 is capable of increasing optical zoom by a factor of two, but this results in a corresponding decrease in FOV size, which is not particularly helpful for the identification of cell or tissue architecture using FNa imaging. However, a zoom function might be advantageous for dyes such as AO or AF (Supplemental Figure 8). Unlike benchtop confocal scanning microscope systems, where zoom can be adjusted in real time (45, 46), handheld CLE systems are limited to a preset zoom because of limitations in miniature design and construction.

Because of the inherent cellular composition of different tumor types, including cellular and regional tissue architectural heterogeneity, CLE system functionality may benefit the surgeon or pathologist by use of a lower resolution and larger FOV image assessment. This situation would allow a closer match to the lower power magnification of a conventional pathology microscope, which would allow the surgeon a larger FOV for scanning the tissue surface (40). However, the portability and capability to move the CLE probe compensate for the smaller FOV, along with Z-stack functionality. Combining multiple small FOV frames together using mosaicking computational algorithms has been shown to yield a larger reconstructed FOV (47).

### Different Concentrations and Timing of FNa Administration

FNa doses in animals are higher than human doses used in brain tumor surgery either with epifluorescence guidance using the operating microscope with a dedicated filter [2–20 mg/kg (48)] or previously used with CLE [5 mg/kg (1)], which most likely is due to faster metabolism of such fluorophores in rodents. CLE imaging likely requires similar or higher, but not lower, dosages than epifluorescent imaging to obtain informative images. CLE imaging was performed within a period of 5 min to 2 h after FNa injection. This timing was chosen to approximate the duration of tumor surgery in humans and complements the previous data on the biodistribution of FNa in a rodent model (30). Overall, the shorter time periods produced a higher number of diagnostic frames, while imaging at longer time periods produced less contrasted images, which corresponds to the findings by Folaron et al. (31).

### Limitations

The current study was performed using a mouse GL261 glioma model. Histologic patterns and the feasibility of FNa imaging with CLE in other experimental tumor models were not addressed in this project. However, clinical experience (1, 7) and the available literature indicate the general feasibility of such an approach for intraoperative brain tumor imaging. The limitations that have not been addressed in this discussion include the need for the development of remote access to the system by another surgeon or neuropathologist or the ability to push images out to cellular devices for rapid assessment or consultation. In addition, the number of images acquired during a case can be very high—in the thousands—and the number of images with artifact caused by motion or other distortions causing them to be unusable is high. Thus, image stabilization control and associated software built into system would be beneficial for quick informative image selection, identification, review, and optimization (49–51).

## Conclusions

Functionality and parameters were assessed for a CLE system (Gen2) designed specifically to perform to the demands and progress of neurosurgery to potentially assist in managing invasive brain tumors in a preclinical setting. Experimental brain tumor models (*in vivo*) and human tumor samples (*ex vivo*) were employed to discern and describe optimal imaging parameters for microscopic histologic visualization with a clinical-grade CLE system and intravenous FNa as a contrast fluorophore. Visualized histologic patterns and pixel intensity values were of comparable quality on Gen1 and Gen2 systems, if not improved with the Gen2 system. The Gen2 had a limitation of a smaller FOV, while having higher resolution, clearer images, and a more responsive user interface, including an autobrightness function and volumetric Z-stack image acquisition that enrich diagnostic possibilities and imaging functionality.

Such portable CLE systems are designed ultimately for *in vivo* use. CLE systems cannot replace the extensive functionality of benchtop confocal scanning microscopes, and they are not designed to accomplish such tasks. CLE systems depend on their specificity, rapid applicability, and portability; their functions, ergonomics, and employment depend on the demands of the surgical or diagnostic specialty. Additionally, the properties of the emitted and detected light range and concomitant fluorophores have a critical impact on use and efficacy.

The CLE provides the neurosurgeon with real-time intraoperative image resolution at the cellular level that is the critical basis of the potential for cell-precision surgery (although neurosurgical instrumentation does not yet provide for precision maneuvers at the cellular level). Although CLE has been used routinely in other medical and surgical specialties, such as for gastrointestinal diagnosis, neurosurgery is in its infancy with respect to CLE use. CLE technology does appear to be responding to the demands of neurosurgeons and neuropathologists for practical introduction into the operating room as an aid in surgery for invasive brain tumors after nearly a decade of developmental exploration and testing.

Concepts for CLE use that must be considered are those that will make the system meaningful in a surgical setting. CLE usefulness to neurosurgery must be proven by being shown to provide a measurable ability to discriminate and guide removal of tumor invasion, thereby extending or optimizing the resection. As much as CLE may provide precise information on where to biopsy or remove tissue, it may be just as crucially used to inform the neurosurgeon where to stop the resection. Further clinical investigation of the diagnostic value of the Gen2 CLE system in fluorescence-guided brain tumor surgery is ongoing.

## Ethics Statement

All patients gave voluntary informed consent as a part of a study protocol approved by the Institutional Review Board of the Barrow Neurological Institute, St. Joseph's Hospital and Medical Center, Phoenix, Arizona.

## Author Contributions

EB, PN, and MP conception and design. EB, EM, AC, AP, and CC acquisition of data, experiments. EB, EM, NM, VB, JE, PN, and MP analysis and interpretation of data. MP, DH, and AS resources, animal model development. MP, PN, and ML supervision. EB, EM, and CC drafting the article. EB, AC, AP, DH, NM, VB, AS, ML, JE, PN, and MP revising article critically for important intellectual content. All authors final approval of the manuscript.

### Conflict of Interest Statement

Carl Zeiss AG had no influence on project design, image acquisition, data interpretation and analysis, or manuscript preparation, and has no agreements for marketing or financial incentive with the Barrow Neurological Institute for the technology in this study. PN has a consultant agreement with Carl Zeiss AG. EB and JE have received travel support from Carl Zeiss AG for attendance at a scientific conference. The remaining authors declare that the research was conducted in the absence of any commercial or financial relationships that could be construed as a potential conflict of interest.

## Abbreviations

5-ALA: 5-aminolevulinic acid
AF: acriflavine
AO: acridine orange
CLE: confocal laser endomicroscopy
DAPI: 4′6-diamidino-2-phenylindole
FNa: fluorescein sodium
FOV: field of view
Gen1: first generation
Gen2: second generation
H&E: hematoxylin and eosin
LSM: laser scanning microscopy
PpIX: protoporphyrin.
