# Supplementary material for: Progress in Confocal Laser Endomicroscopy for Neurosurgery and Technical Nuances for Brain Tumor Imaging With Fluorescein
Source: Front Oncol. 2019 Jul 3;9:554. doi: 10.3389/fonc.2019.00554 (PMC6616132; doi:10.3389/fonc.2019.00554)
Supplement: Supplementary file 1 [file Table_1.docx]

**SUPPLEMENTARY TABLE**

**Supplementary Table 1.** Comparison of image acquisition speed.

| **Image size, pixels** | **Gen1** | **Gen2** |
| --- | --- | --- |
| 1920 × 1080 | N/A | 1.29 spf |
|  | N/A | 0.78 fps |
| 1024 × 1024 | 1.2 spf | N/A |
|  | 0.83 fps | N/A |
| 1920 × 540 | N/A | 0.71 spf |
|  | N/A | 1.41 fps |
| 1024 × 512 | 0.8 spf | N/A |
|  | 1.25 fps | N/A |
| 1920 × 270 | N/A | 0.44 spf |
|  | N/A | 2.27 fps |
| 1920 × 135 | N/A | 0.26 spf |
|  | N/A | 3.79 fps |

Abbreviations: fps, frames per second; N/A, not available; spf, seconds per frame.
