# Supplementary material for: Progress in Confocal Laser Endomicroscopy for Neurosurgery and Technical Nuances for Brain Tumor Imaging With Fluorescein
Source: Front Oncol. 2019 Jul 3;9:554. doi: 10.3389/fonc.2019.00554 (PMC6616132; doi:10.3389/fonc.2019.00554)
Supplement: Supplementary file 2 [file Image_1.pdf]

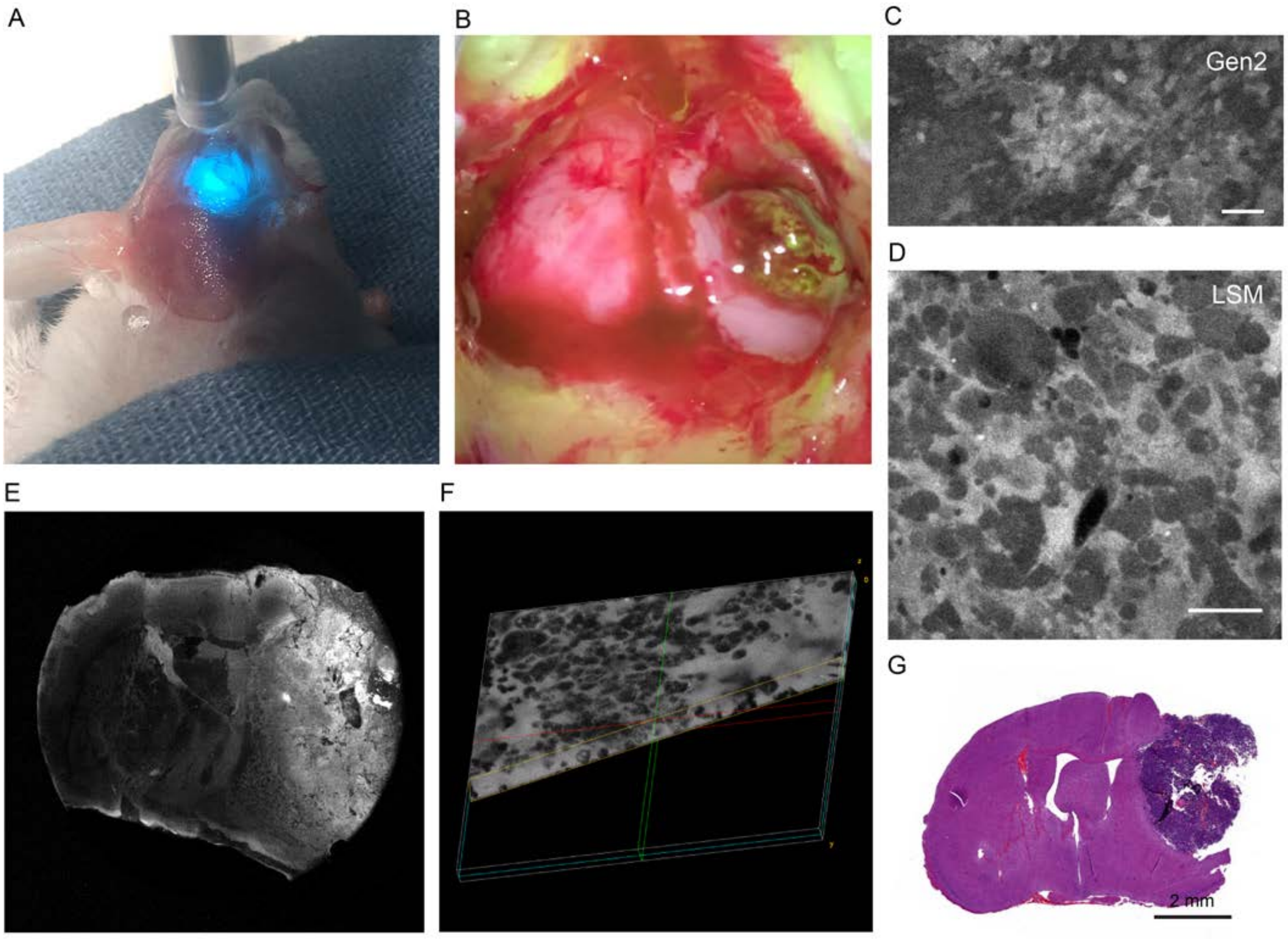

Supplemental Figure 1. Overview of the study methods. **(A)** In vivo and rapid ex vivo confocal laser endomicroscopy (CLE) imaging. **(B)** Pentero 900 imaging with Yellow 560 filter. **(C)** Generation 2 (Gen2) CLE image acquisition. Scale bar is 50 µm. **(D, E)** Benchtop laser scanning microscopy (LSM). Scale bar is 50 µm. **(F)** Z-stack volumetric reconstruction sliced obliquely to demonstrate the thickness of the stack. **(G)** Hematoxylin and eosin stained coronal brain slice.
Used with permission from Barrow Neurological Institute, Phoenix, Arizona.

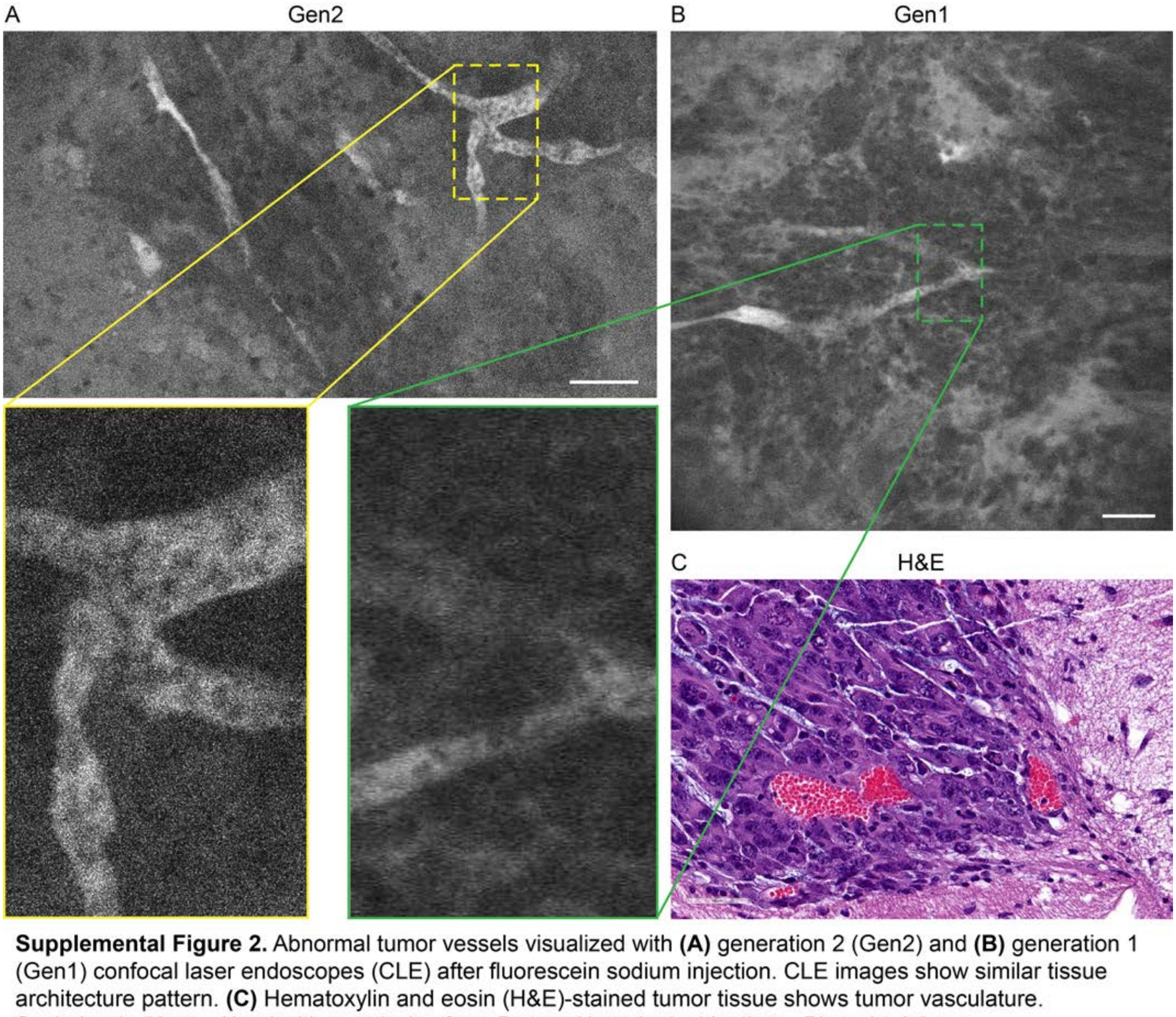

Supplemental Figure 2. Abnormal tumor vessels visualized with **(A)** generation 2 (Gen2) and **(B)** generation 1 (Gen1) confocal laser endoscopes (CLE) after fluorescein sodium injection. CLE images show similar tissue architecture pattern. **(C)** Hematoxylin and eosin (H&E)-stained tumor tissue shows tumor vasculature. Scale bar is 50 µm. *Used with permission from Barrow Neurological Institute, Phoenix, Arizona.*

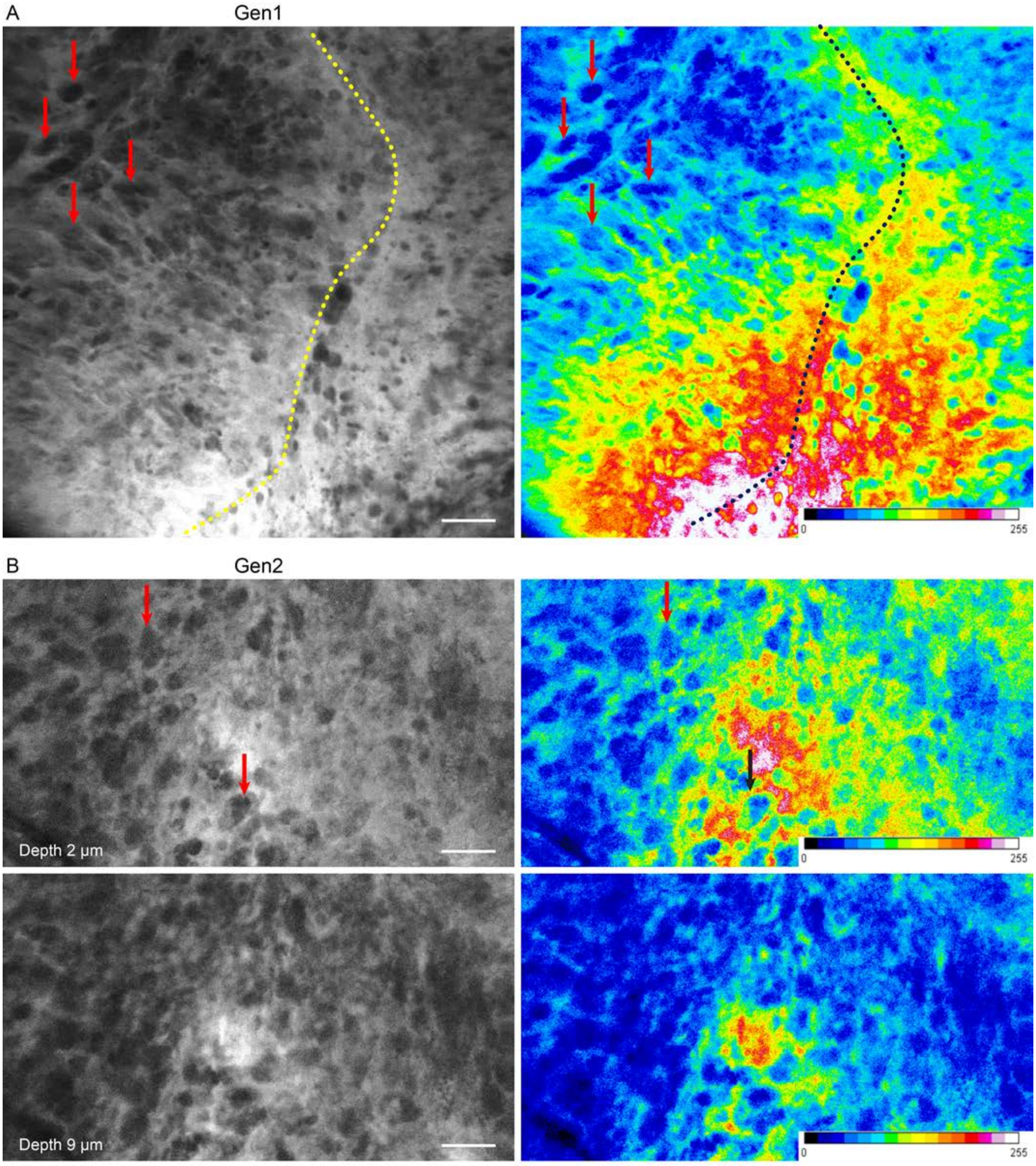

Supplemental Figure 3. Fluorescein sodium diffusion gradient from the vessel into the tumor. Images acquired with **(A)** generation 1 (Gen1) and **(B)** generation 2 (Gen2) confocal laser endomicroscopes. Arrows point to the individual tumor cells. The bright area shown in red represents the area close to the vessel. Dotted line shows tumor border, with tumor located to the left. Scale bar is 50 µm.
Used with permission from Barrow Neurological Institute, Phoenix, Arizona.

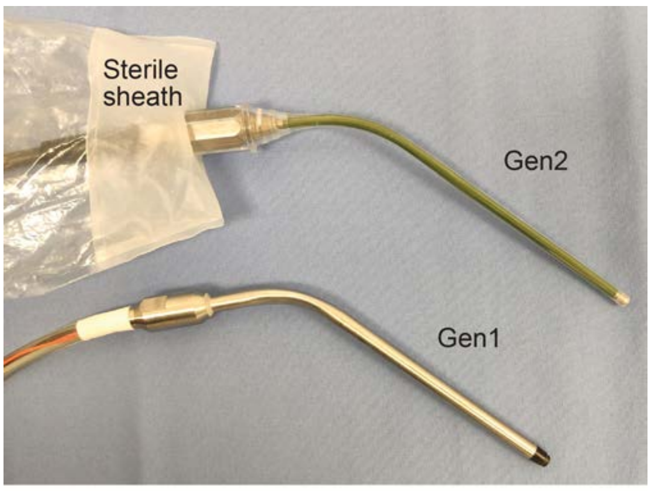

Supplemental Figure 4. Generation 1 (Gen1) and generation 2 (Gen2) confocal laser endomicroscopes.
Used with permission from Barrow Neurological Institute, Phoenix, Arizona.

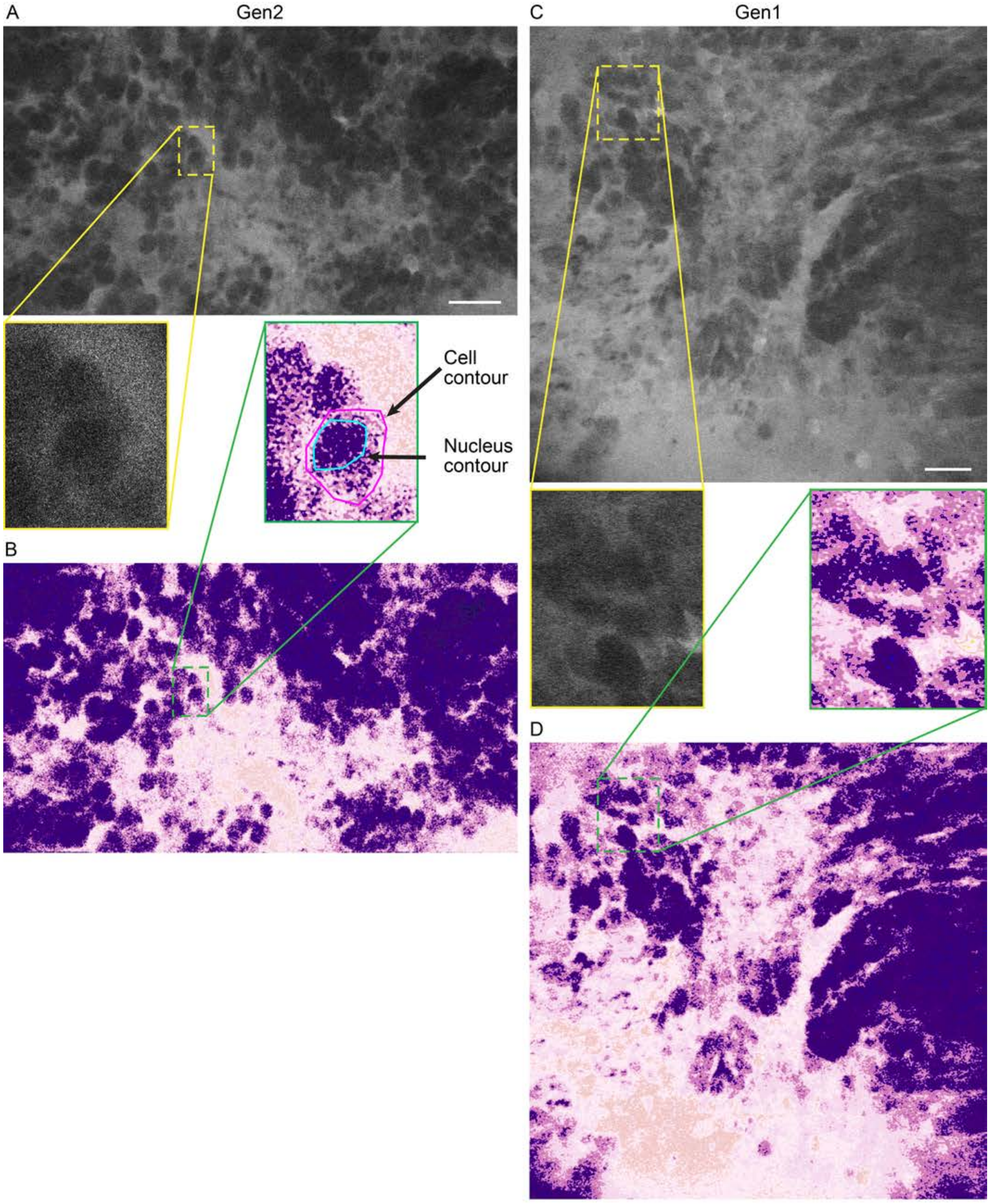

Supplemental Figure 5. Fluorescein dye delineating nuclei and cellular silhouettes in brain tumor, as visualized by generation 1 (Gen1) and generation 2 (Gen2) confocal laser endomicroscopes (CLE). Images from **(A)** Gen2 and **(C)** Gen1 show how the gradient of fluorescein sodium penetration through the biologic membranes helps delineate nuclei and cellular silhouettes in the animal brain tumor region. Image analysis performed in FIJI. Selected regions of interest are cropped, and filters are applied: despeckle, smooth, average 2 pixels. A custom color look-up table is applied on a grayscale image. Brightness was increased for better visualization of contrast between the gray tones. **(B)** and **(D)** are colored CLE images from Gen2 and Gen1 systems, respectively, in the style of a hematoxylin and eosin stain, to illustrate the similarity in quality between CLE imaging and histology. **(A)** and **(B)** are corresponding images. **(C)** and **(D)** are also corresponding images. Scale bar is 50 μm.
Used with permission from Barrow Neurological Institute, Phoenix, Arizona.

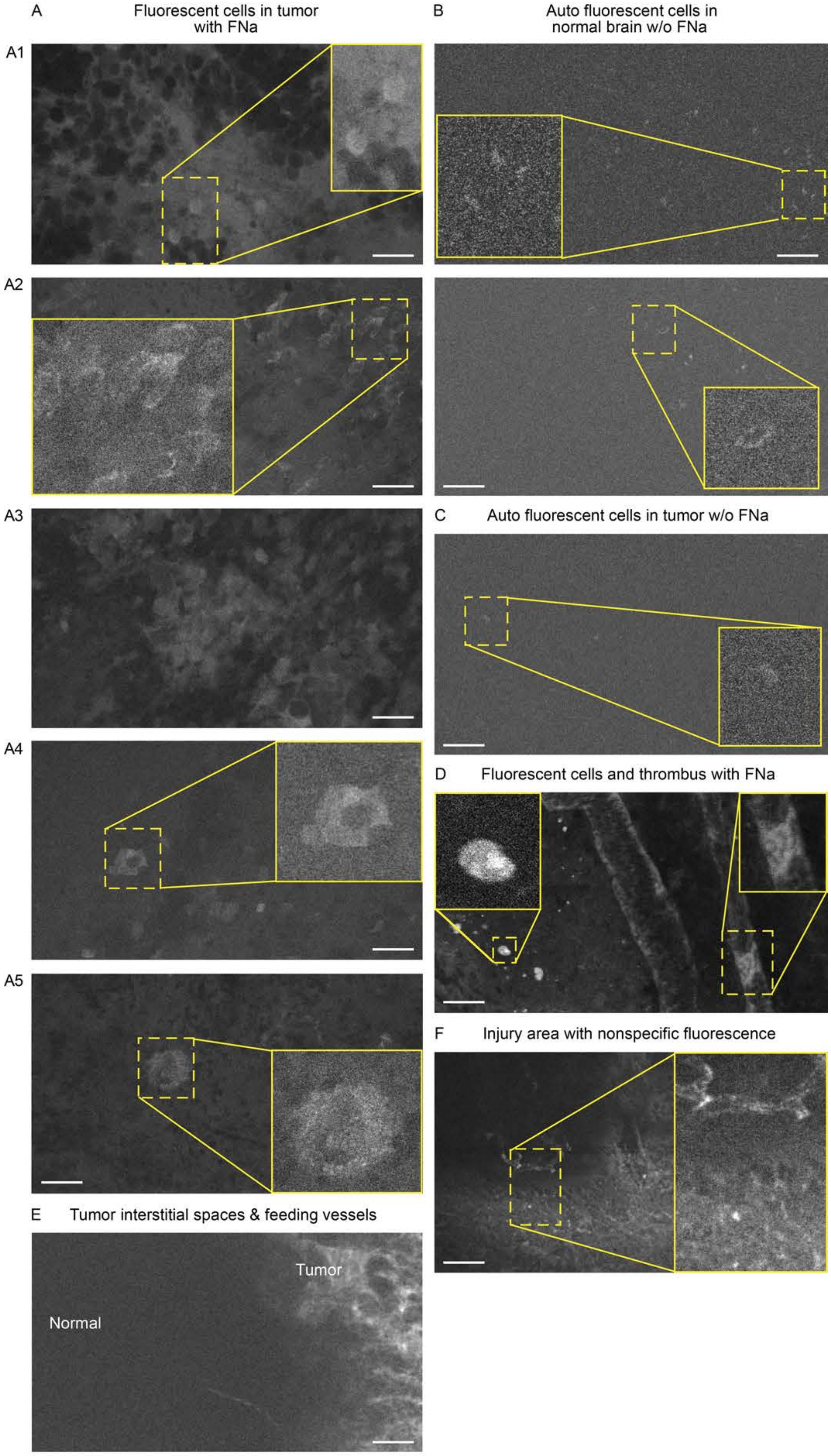

Supplemental Figure 6. Various patterns of fluorescence visualized with generation 2 confocal laser endomicroscope. **(A)** Some tumor cells exhibited fluorescein sodium (FNa) fluorescence. **(A1)** Singular fluorescent tumor cells. **(A2)** Multiple tumor cells with granular fluorescent pattern. **(A3)** Mix of dark nonfluorescent and bright fluorescent cells. **(A4 and A5)** Giant fluorescent cells with a diameter of about 50 µm. **(B)** Examples of sparse autofluorescence. Some cells just have inclusions that are visible; in others a dark nucleus may be distinguished. Note smaller cell size. **(C)** Some cells from tumor also contain autofluorescent inclusions. **(D)** Individual parenchymal fluorescent cells and intravascular thrombus with visible fluorescent cells are visible. **(E)** FNa is visible in the vessels of the normal brain and in the interstitial spaces of the tumor area. **(F)** Injured nontumor brain tissue embedded with FNa; some normal vessels are also visible. Scale bar is 50 µm. *Used with permission from Barrow Neurological Institute, Phoenix, Arizona.*

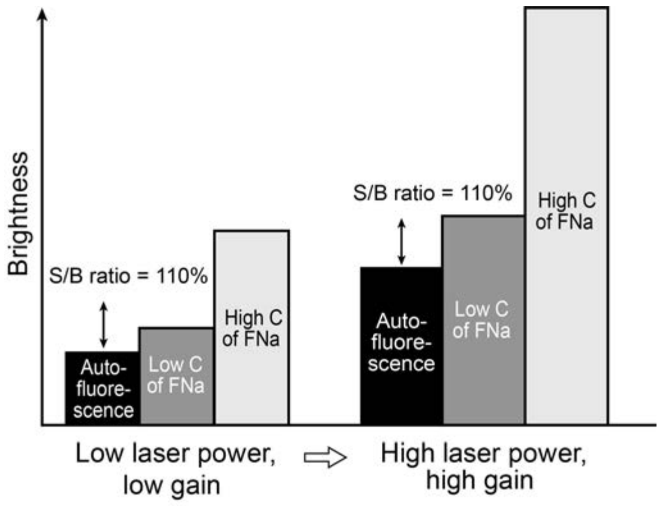

Supplemental Figure 7. Relationship between gain, laser power, and contrast, in terms of brightness. Increase in gain or laser power has limited impact on contrast because of the proportional increase of autofluorescence and non-specific fluorescein sodium signal in the same spectral range. Therefore, relative signal to background (S/B) ratio stays constant. C = concentration; FNa = fluorescein sodium. Scale bar is 50 μm. *Used with permission from Barrow Neurological Institute, Phoenix, Arizona.*

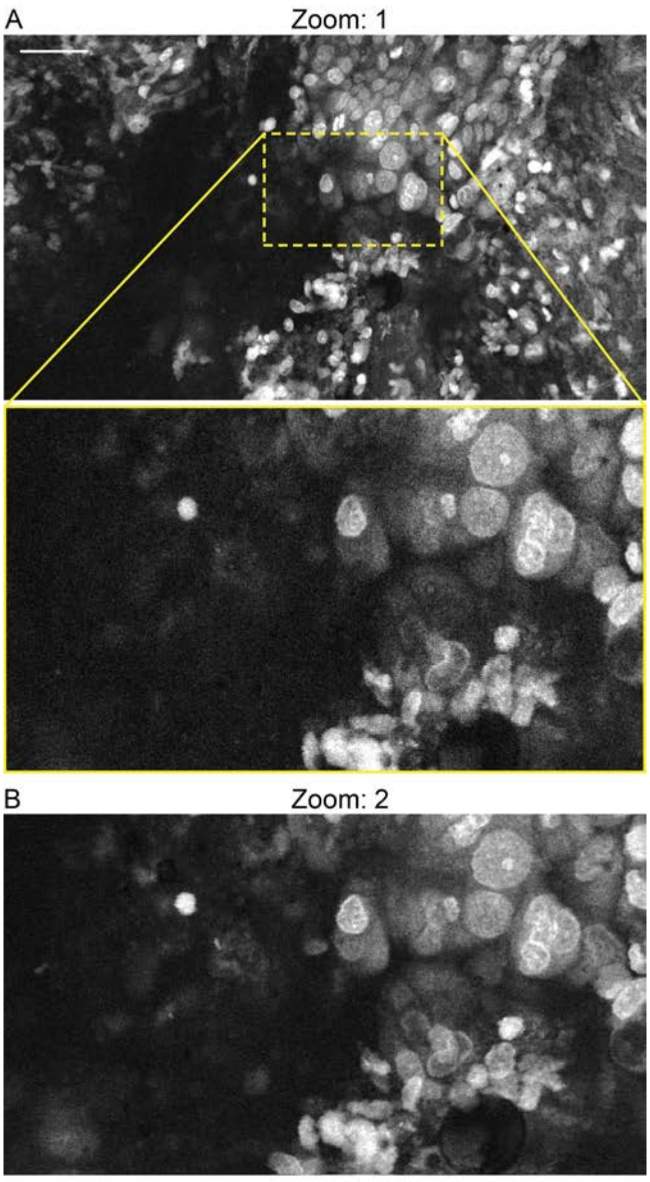

Supplemental Figure 8. Comparison of generation 2 confocal laser endomicroscope images taken with various zoom levels. Human samples stained with acridine orange are used for analysis and illustration. **(A)** 1× zoom provides similar image quality to **(B)** 2× zoom. Higher zoom shows smaller field of view enlarged on the screen. Scale bar is 50 μm. *Used with permission from Barrow Neurological Institute, Phoenix, Arizona.*
